# Comparison of tumor curettage and resection for treatment of giant cell tumor of the bone around the knee joint

**DOI:** 10.12669/pjms.323.9654

**Published:** 2016

**Authors:** Sheng Zhang, Jianhua Zhang, Xin Wang

**Affiliations:** 1 Sheng Zhang, Department of Orthopedics, Yan’an People’s Hospital, Yan’an 716000, Shanxi Province, China; 2 Jianhua Zhang, Department of Orthopedics, the Affiliated Hospital of Yan’an University, Yan’an 716000, Shanxi Province, China; 3 Xin Wang, Department of Orthopedics, Yan’an People’s Hospital, Yan’an 716000, Shanxi Province, China

**Keywords:** Giant cell tumor of bone around knee joint, Tumor resection, Enneking scoring, Tumor curettage

## Abstract

**Objective:**

To analyze the efficacies of tumor curettage and resection for treatment of giant cell tumor of the bone (GCTB) around the knee joint (KJ).

**Methods:**

A total of 126 KJ-GCTB cases were treated at our department from August 2011 to February 2015. These cases were divided into two groups (A and B) according to treatment methods. Group A underwent tumor curettage, while group B underwent tumor resection.

**Results:**

The relapse rates did not differ significantly between the groups (*P*>0.05), while the complication rate in group A was significantly lower than that in group B (*P*<0.05). In addition, the Enneking score for group A was significantly higher than that for group B (*P*<0.05); in addition, postoperative local recurrence, histopathological grading according to Jaffe, and radiographic imaging-based Campanacci’s staging positively correlated (*P*<0.05).

**Conclusion:**

Tumor curettage was the preferred surgical approach for patients with KJ-GCTB.

## INTRODUCTION

Giant cell tumor of the bone (GCTB) was first described by Jaffe in 1940 as one of the common primary bone tumors; while its source remained uncertain, it may originate from the mesenchymal tissues of the bone marrow.^1^ GCTB has aggressive features, with huge corrosive destruction of the sclerotin, which left rare tendency of reactive new bone formation and healing; However, GCTB may also penetrate the cortical bone and form soft tissue masses, leading to high recurrence rates after curettage. Several cases have been reported to exhibit partial canceration or lung metastasis (so-called benign metastasis).^2^,^3^

GCTB is typically considered to have low malignant potential. This disease commonly occurs in individuals aged between 20’s to 50’s, and is more common in women than in men.^4^ Although the epiphysis is the primary site of GCTB, the lesions might gradually expand to invade the metaphysic.^5^ GCTB most commonly affects the long bones, primarily the inferior femoral and anterior tibial ends. GCTB is one of the common orthopedic tumors, accounting for 10 to 20% of primary bone tumors,^6^ most commonly at the ends of the long bone. Although it is normally benign, it also has the tendency develop into cancer.^7^,^8^

A variety of clinical treatment methods is used for GCTB, among which tumor curettage and tumor resection are the most common.^9^ Tumor resection can completely remove the tumor and prevent recurrence, while tumor curettage can retain joint functions.^10^ The current study evaluated the efficacies of tumor curettage and tumor resection of the knee joint (KJ)-GCTB.

## METHODS

### General information

**Fig.1:**
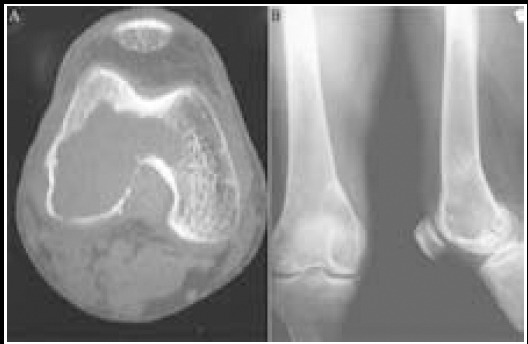
X-ray film of KJ-GCTB.

**Fig.2:**
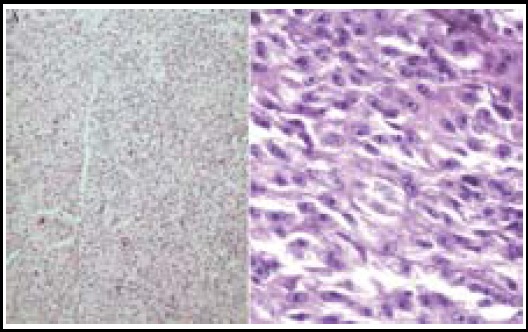
HE staining of KJ-GCTB.

A total of 126 KJ-GCTB cases were treated at our department from August 2011 to February 2015. The patients included in the present study underwent imaging examinations (Fig.1), and they were diagnosed with GCTB by biopsy evaluation (Fig.2). These patients were divided into two groups, A (76 cases) and B (50 cases), which underwent different surgical treatments. Group A had 43 men and 34 women with a mean age of 36.4±10.2 years. Among these cases, 42 and 34 exhibited tumors on the anterior tibial segment and inferior femoral segment, respectively. A histopathological examination according to Jaffe revealed 17 grade I, 40 grade II, and 19 grade III cases.

Group B had 28 men and 22 women, with a mean age of 35.9±9.3 years. Among them were 28 cases with tumor on the anterior tibial segment and 22 cases with tumor on the inferior femoral segment. The histopathological examination according to Jaffe revealed nine grade I, 27 grade II, and 14 grade III cases. The general characteristics, including age, sex, and tumor types, did not differ significantly between the two treatment groups (*P*>0.05). This study was conducted in accordance with the declaration of Helsinki after approval from the Ethics Committee of Yan’an People’s Hospital. Written informed consent was obtained from all participants.

### Patient treatments

All patients underwent surgery under lumbar anesthesia in the supine position. Group A underwent tumor curettage after determining the location and size of the windowing before surgery; the windowing focuses on the sites with the most serious bone destruction, and tumor tissues were washed with carbolic acid (Suzhou Dinghong Chemical Company, Shanghai, China) to inactivate the tumor cells. The degree of bone impairment was assessed, and artificial and allogeneic bone transplantation was performed for internal fixation in cases where the impairment might have affected the supporting role of the bone scaffold, compacted the graft, and covered the anterior support. If the affected site was close to the articular surface, the sub-articular surface was also filled simultaneously, and allogeneic bone with the cortical bone was used to cover the affected site in order to ensure cortical bone continuity.

Group B underwent tumor resection, which is often used to treat patients with extensive bone destruction; the resection was performed in accordance with the standards of wide excision of bone tumor, and allogeneic bone with large articular surface was used for repair.

### Outcome measures

All patients were followed up for more than six months; the treatment efficacies, relapse rates, and treatment risk factors between the two groups were analyzed. The postoperative functions were scored using the Enneking scoring system.

### Statistical analysis

SPSS Statistics for Windows, version 17.0 was used for statistical analysis of the data, and postoperative Enneking scores were expressed as±s and analyzed with t tests. Postoperative recurrence and complication rates were expressed as % and analyzed by χ^2^ test, and factors associated with treatment efficacies of GCTB were analyzed by logistic regression analysis, with *P*<0.05 considered statistically significant.

## RESULTS

### Postoperative recurrence rates

Group A had 22 cases of postoperative recurrence, corresponding to a recurrence rate of 25.3%. In comparison, while group B had 10 cases of postoperative recurrence and a recurrence rate of 20.0%, the recurrence rates did not differ significantly between the two groups (*P*>0.05).

### Postoperative complications

**Fig.3:**
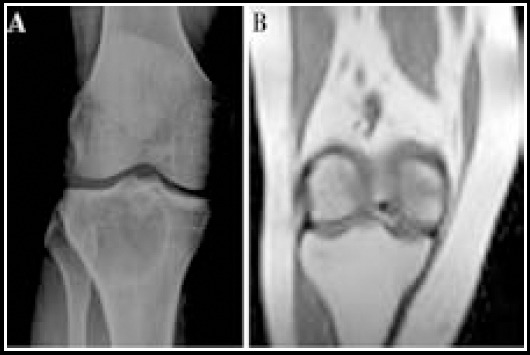
X-ray pictures from each group of A and B patients. A. Line giant cell tumors of bone tumors around the knee joint after shaving x-ray; B. Around the knee joint line giant cell tumors of bone tumor resection of the large X-ray.

Group B had four cases of bacterial infection, three cases of graft reaction, and a complication rate of 14.0%. Group A had one case of bacterial infection and one case of graft reaction, corresponding to complication rate of 2.3%. The complication rate in group A was significantly lower than that in group B (Fig.3, *P*<0.05).

### Postoperative Enneking scores

The average Enneking score for group A (29.16±8.53), was significantly higher than that for group B (19.22±7.43) (*P*<0.05).

### Analysis of impacting factors

Age, sex, onset time, and pathological grading were included into the statistics for the analysis; the analysis revealed that postoperative local recurrence, Jaffe histopathological grade (r=3.73) and tumor radial Campanacci’s stage (r=4.06) were positively correlated (*P*<0.05).

## DISCUSSION

GCTB was first described in 1818. While it was originally considered a benign lesion, recent studies^11^ suggest that this tumor occurs commonly, typically at the distal femur and proximal tibia around the knee joints; it has aggressive features, and it is considered a potentially malignant tumor that could destroy the sclerotin around the knee joint and affect knee function. The current treatment methods mainly include surgical approaches, while related surgical methods remain controversial;^12^ therefore, analysis of the efficacies of tumor curettage and tumor resection for treatment of KJ-GCTB has practical significance.

Tumor curettage and resection are two approaches often used for treatment of KJ-GCTB. Because tumor curettage retains the patient’s own cartilage surface and bone scaffold^13^ as well as the knee function, it is one of the basic surgical treatments often and widely used for KJ-GCTB. However, many studies have reported high relapse rates ranging from 27 to 50%;^13^ in the present study, group A had 22 cases of recurrence, a 25.3% rate consistent with a previous study.^14^ Tumor resection is also used often for the treatment of KJ-GCTB. It can completely remove the tumors and prevent recurrence, thus reducing the postoperative recurrence rate. In the present study, group B had 10 cases of recurrence and a 20.0% recurrence rate, which was lower than that of group A; however, this difference was not statistically significant (*P*>0.05). Previous studies^15^,^16^ have reported increased long-term complications with tumor resection, similar to the findings of the present study. In the present study, group B had four cases of bacterial infections and three cases of graft reaction, corresponding to a complication rate of 14.0%. Group A had one case each of bacterial infection and graft reaction, corresponding to a complication rate of 2.3%. The incidence of complications in group A was significantly lower than that in group B (*P*<0.05). Therefore, based on these findings, patients without aggressive features should undergo tumor curettage, while those with severe bone destruction, or when reconstruction after tumor curettage cannot meet the patient’s loading requirements, should undergo tumor resection.

Previous studies on tumor curettage have identified the windowing position to be critical, as the window should be large enough to adequately support the patient’s bone defects and be convenient for surgical procedures. The current methods for filling bone defects include autologous, allogeneic, and artificial bone, etc.; while autologous bone is currently the preferred choice, many patients have large bone defects for which the autologous bone is inadequate. In these situations, an allogeneic or artificial bone could accelerate bone repair.^17^-^19^ In this group, tumor curettage may cause significant bone defects, with an increased demand for filling materials that an autogenous bone could not satisfy. Bone cement is often used, as it is relatively cheap and the heat generated during polymerization may inactivate the tumor cells;^20^ however, a number of studies have also suggested that bone cement might lead to a bone loss or reduced bone healing. Therefore, the present study used a combination of autologous and allogeneic bone transplantation for reconstruction. Post-tumor resection reconstruction normally involves the use of allogenic bone graft or prosthesis replacement therapy, which has relatively higher incidence of complications and requires a long healing process. The results showed that the incidence of complications in group A (2.3%) was significantly lower than that in group B (14.0%) (*P*<0.05), and follow-up examinations revealed that the joint function scores of group B were significantly lower than those of group A, suggesting that tumor curettage had more pronounced treatment effects. The method for treatment of KJ-GCTB is generally selected according to patient specific performance and imaging findings.^21^ One study reported that the therapeutic effects of KJ-GCTB treatments were positively correlated with Jeffer’s grades.^22^ In the present study, patient outcomes were analyzed by logistic regression analysis; the results showed that postoperative local recurrence, Jaffe histopathological grade (r=3.73), and radiographic Campanacci stage (r=4.06) were positively correlated (*P*<0.05). Therefore, patients with Jeffer’s pathological grade I, as well as tumor size and bone diameter less than 1/2 with relatively mild clinical manifestations should undergo tumor curettage. Similarly, patients with Jeffer’s pathological grade III and radiographic Campacci’s stage III, with relatively significant cancer-associated damage and whose cortical bone is easily invaded, should undergo tumor resection.

In short, a reasonable surgical approach for treatment of KJ-GCTB should consider the tumor location, the degree of damage, and range of the tumor. Tumor curettage offers effective tumor removal without affecting supporting bone. Therefore, this method should be considered the preferred surgical approach, while tumor curettage should be considered for patients with severe bone destruction, or for whom the loading requirements could not be met after reconstruction.

In the treatment of patients with giant cell tumor around the knee joint one should select the right surgical method. Tumors curettage can not affect bone support effective removal of the tumor, surgery is the preferred way. The main defect is that their tumor recurrence rate is higher. After tumor resection of large lesions, the activity of the joints is poor, but the postoperative local recurrence rate is low. Hence keeping in view the actual situation one should choose the right surgical procedure.
